# The comparative study of color doppler flow imaging, superb microvascular imaging, contrast-enhanced ultrasound micro flow imaging in blood flow analysis of solid renal mass

**DOI:** 10.1186/s40644-022-00458-2

**Published:** 2022-05-03

**Authors:** Yiran Mao, Jie Mu, Jing Zhao, Fan Yang, Lihui Zhao

**Affiliations:** 1 Department of Ultrasound, Tianjin Medical University Cancer Institute and Hospital, National Clinical Research Center for Cancer, Tianjin, China; 2 Key Laboratory of Cancer Prevention and Therapy, Tianjin’s Clinical Research Center for Cancer, Tianjin, China

**Keywords:** Ultrasound, Renal tumor, SMI, MFI

## Abstract

**Purposes:**

To evaluate the value of Color Doppler Flow Imaging (CDFI), Superb Microvascular Imaging (SMI) and Contrast-enhanced Ultrasound Microflow Imaging (MFI) in display the microvascular blood flow signals in renal solid lesions.

**Methods:**

142 patients with 144 renal masses were examined by CDFI, SMI and MFI simultaneously. We compared the difference of blood flow grading and vascular architecture based on CDFI, SMI and MFI.

**Results:**

The blood flow signals detection rates of CDFI, SMI and MFI were 78.5% (113/144), 88.9% (128/144) and 93.8% (135/144), respectively. Concentrated on blood flow grading, The coincidence rates of CDFI and SMI were 64.58% (93/144) and 81.25% (117/144) referring to MFI, respectively. Blood flow grade 2–3 in CDFI is significantly lower than SMI(*x*^*2*^ = 5.557, *P* = 0.018) and MFI (*x*^*2*^ = 10.165, *P* = 0.001). Whereas there was no significant difference between SMI and MFI (*x*^*2*^ = 2.372, *P* = 0.499). Concentrated on vascular architecture, the coincidence rates of CDFI and SMI were 56.25% (81/144) and 75.69% (109/144) referring to MFI, respectively. Vascular architecture type IV and V in CDFI was significantly lower than SMI (*x*^*2*^ = 18.217, *P* < 0.001) and MFI (*x*^*2*^ = 29.518, *P* < 0.001). Whereas there was no significant difference between SMI and MFI (*x*^*2*^ = 3.048, *P* = 0.550). The sensitivity and specificity of CDFI, SMI and MFI in the diagnosis of renal mass were 61.29% and 90.20%, 79.57% and 88.24%, 88.17% and 84.31% respectively. The areas under the ROC curve of the three were 0.757, 0.839 and 0.862, respectively. There was a statistically significant difference between CDFI and MFI (Z = 3.687, *P* = 0.0002), while there was no statistically significant difference between SMI and MFI (Z = 1.167, *P* = 0.2431).

**Conclusion:**

SMI and MFI are superior to CDFI in showing blood flow signals in renal solid masses, and it can perform blood flow and vascular architecture more accurately.

**Advances in knowledge:**

SMI is similar to MFI in its ability to display fine vessels and diagnostic efficiency, and has application value in the diagnosis and differential diagnosis of renal solid masses.

Renal cancer is one of common malignant tumors. Due to the similar characteristics of imaging features of benign and malignant renal tumors, the application of conventional ultrasound and CT for diagnosis has certain limitations, which may easily lead to un-diagnosis or misdiagnosis [1]. Blood flow information plays an auxiliary role in distinguish the benign and malignant tumor. Superb microvascular imaging (SMI) is a new color Doppler technology, which can reduce the influence of motion clutter, improve the detection rate of low-speed blood flow signals, and display tiny vessels [2]. In this study, Color Doppler Flow Imaging (CDFI) of 144 renal masses was performed. CDFI, SMI and contrast-enhanced ultrasound micro flow imaging (MFI) images were analyzed to explore the ability of these three methods in evaluating the blood flow information of renal masses.

## Materials and methods

### Patients

From December 2017 to March 2019, a total of 315 patients with renal masses were found by ultrasound examination in our hospital. Exclusion criteria were as follows: (1) Renal cystic or cystic solid masses; (2) CDFI, SMI and MFI tests are not carried out; (3) Pathological results had not obtained; (4) unable to cooperate with the examination because of suffering from serious heart and lung disease; (5) Puncture or chemoradiotherapy were performed before ultrasound. Finally, 144 masses were selected from 142 patients. There were 140 cases of single disease and 2 cases of double disease, including 89 males and 53 females, aged from 24 to 80 years old, with an average of (56.75 ± 9.42) years old.

This study was approved by the ethics committee of Tianjin Medical University Cancer Institute and Hospital. Written consents were obtained from each patient.

### Equipment and examination

All US, CDFI, MFI and SMI examinations were performed with an Aplio 500 US system (Toshiba America Medical Systems Inc, USA) equipped with a 11–15 MHz linear array transducer. Ultrasound contrast agent was Sonovue from Bracco, Italy, and the contrast microvesicles were phospholipid-coated sulfur hexafluoride (SF6) with an average diameter of 2.5um. Informed consent is signed by all study participants.

#### CDFI and SMI

Routine gray scale ultrasound was performed supine after abdomen exposed fully. The size, location and morphology of the lesion were observed. CDFI mode and SMI mode were applied to observe blood flow in the tumor on multiple sections. Instructing patients to hold breath during examination; The sampling gain contained the tumor and its surrounding area of about 1 cm. The color gain was adjusted to show small blood vessels without artifact. The SMI range was set to 1.2 ~ 4.7 cm/s. The same section was selected for CDFI and SMI images. Synchronous storage of static and dynamic images, video coexist disk were performed.

#### MFI method

The contrast medium was injected with 5 mL normal saline after Shaked and mixed. 0.8 ~ 1.5 ml of the configured contrast agent, which was extracted and injected into the cubital vein (bolus injection), followed by a rapid injection of 5 mL normal saline to observe the tumor angiographic performance. Imaging parameters: mechanical index < 0.2; Dynamic range of 50 ~ 60 dB; Contrast harmonic imaging gain 55 dB. When the contrast agent entered the clearance period, pressed the FLASH button to conduct the explosion, started the MFI mode, observed the imaging track of the microbubble in the tumor microvessel, and save synchronizing video. In order to reduce the influence of contrast agent on CDFI and SMI examination, MFI examination were performed after CDFI and SMI examination.

A single radiologist with more than 10 years of experience in US performed all of the scans. Another two ultrasound radiologists with more than 10 years of experience in US diagnosis reviewed all of the scans in blind to each other.

#### Blood flow grade

Blood flow information analysis of tumor included vascular architecture and blood flow grade. The blood flow signal was graded by Adler method: Grade 0, no blood flow in the lesion; Grade 1, small amount of blood flow, 1 to 2 point or fine rod-shaped vessels in the lesion; Grade 2, medium blood flow, 3 ~ 4 punctate vessels or 1 important vessel, the length of which can be close to or beyond the radius of the lesion; Grade 3, rich blood flow, more than 5 punctate vessels or 2 long vessels [3], shown in Table 1.

**Table 1:** The definition of Blood flow grade and Vascular architecture classification

**Blood flow grade**		
Grade	definition	description
I	small amount of blood flow	1 to 2 point or fine rod-shaped vessels in the lesion
II	medium blood flow	3 ~ 4 punctate vessels or 1 important vessel, the length of which can be close to or beyond the radius of the lesion
III	rich blood flow	more than 5 punctate vessels or 2 long vessels
**Vascular architecture classification**		
Type	definition	description
I	no blood flow	no blood flow signal was detected in the tumor
II	stellate blood flow	the blood flow signal inside the tumor is rare, showing stellate distribution
III	low blood flow	with small blood vessel implantation in the tumor, but rarely with blood flow signal beyond the center of the renal tumor
IV	universal blood flow	there is circumferential blood flow around the tumor and a little blood implantation can be seen inside
V	multi-blood flow	there are blood vessels around the tumor, and multiple or intermittent and relatively thick blood flow signals can be seen inside

#### Vascular architecture classification

Type I: no blood flow signal was detected in the tumor. Type II: stellate blood flow, the blood flow signal inside the tumor is rare, showing stellate distribution; Type III: Low blood flow, with small blood vessel implantation in the tumor, but rarely with blood flow signal beyond the center of the renal tumor; Type IV: Universal blood flow. There is circumferential blood flow around the tumor and a little blood implantation can be seen inside. Type V: multi-blood flow type. There are blood vessels around the tumor, and multiple or intermittent and relatively thick blood flow signals can be seen inside [4], shown in Table 1.

### Statistical method

Data were analyzed using SPSS 22.0 software. The two doctors used Kappa consistency test for the consistency of vascular architecture classification and blood flow grading. Chi-square test and Fisher's exact test were used to compare the classification variables. *P* < 0.05 was considered statistically significant.

## Results

### Ultrasound features

The maximum diameter of the mass ranged from 1.2 to 13.9 cm, with an average of (4.45 ± 2.02) cm. There were 85 lesions with maximum diameter ≤ 3 cm and 59 lesions > 3 cm. 62 on the left and 82 on the right; There were 92 hypoechoic, 18 isoechoic and 24 hyperechoic. 61 had clear boundaries and 83 had unclear boundaries.

### Pathological results

Among 144 solid renal masses, 51 were benign lesions, including 46 angiomyolipomas, 4 eosinophilic adenomas, and 1 inflammatory pseudotumor. There were 93 malignant lesions, including 68 clear cell carcinoma of the kidney, 13 chromophobe cell carcinoma, 10 papillary renal cell carcinoma, 1 mucinous carcinoma, and 1 nephroblastic carcinoma.

### The consistency of blood flow grade and vascular architecture of renal lesions

According to two single ultrasound radiologists’ results, the Kappa coefficients of renal mass blood flow grading in CDFI, SMI and MFI modes were 0.731, 0.856 and 0.865, respectively, and the Kappa coefficients of blood vessel architecture were 0.772, 0.819 and 0.793, respectively.

### Blood flow grading analysis of 144 renal lesions based on CDFI, SMI and MFI

**Fig. 1:**
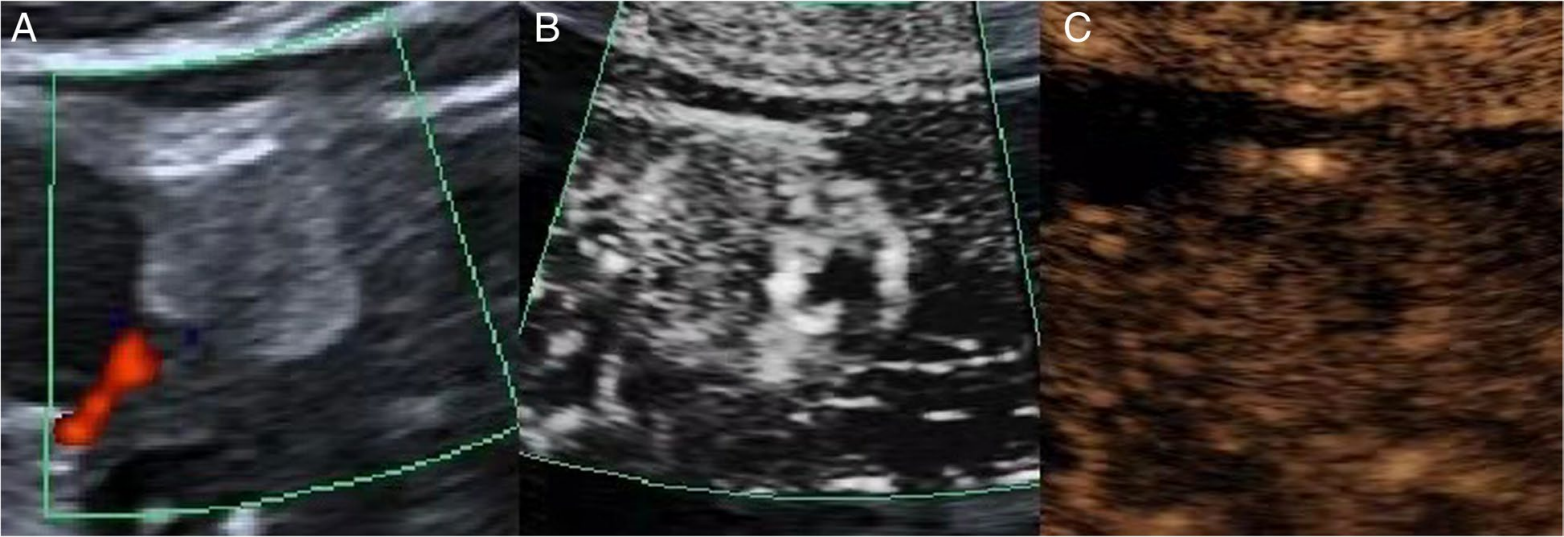
A solid mass of left kidney **A** ultrasound imaging of CDFI, hyperechoic mass with clear boundary, regular shape, and inhomogeneous internal echo **B** SMI show point-line blood flow signal in the tumor, blood flow grade 1. **C** MFI show point-line blood flow signal in the tumor, blood flow grade 1, pathological result was hamartoma

**Fig. 2:**
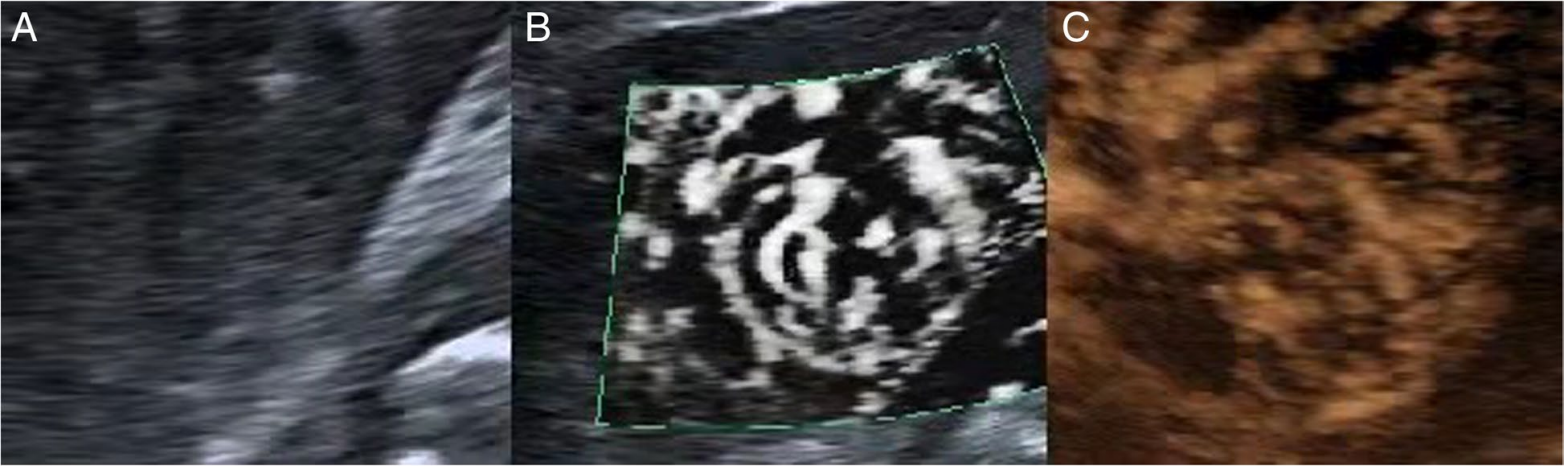
A solid tumor of the right kidney **A** ultrasound imaging, hypoechoic mass with unclear boundary, regular shape, and inhomogeneous internal echo **B** SMI show multiple large vessels and their dot and linear blood flow branches around and inside the tumor, blood flow grade 3 **C** MFI ultrasonography showed more clearly vascular branches than SMI, and it could show the branch shape. blood flow grade 3. The pathological result was clear cell renal cell carcinoma

The blood flow detection rates of CDFI, SMI and MFI were 78.5% (113/144), 88.9% (128/144) and 93.8% (135/144), respectively. The blood flow grading results were shown in Table 2. Based on CDFI, SMI and MFI, Blood flow grading from 0 to 1 was 56.3% (81/144), 42.3% (61/144) and 37.5% (54/144), respectively. Blood flow grade 2 to 3 were 43.8% (63/144), 57.6% (83/144), and 62.5% (90/144), respectively. Using MFI images in clearance period as a reference, the coincidence rates of CDFI and SMI blood flow grading were 64.58% (93/144) and 81.25% (117/144), respectively. MFI and SMI can detect small, low-speed blood flow signals which CDFI cannot display (Figs. 1 and 2).

**Table 2:** Blood flow grade of 144 renal lesions based on CDFI, SMI and MFI

	CDFI				SMI				MFI			
	0	1	2	3	0	1	2	3	0	1	2	3
Benign	18	28	3	2	11	32	4	4	7	34	4	6
angiomyolipomas	17	25	3	1	10	32	2	2	6	34	2	4
eosinophilic adenomas	0	3	0	1	0	0	2	2	0	0	2	2
inflammatory pseudotumor	1	0	0	0	1	0	0	0	1	0	0	0
Malignance	13	22	23	35	5	13	23	52	2	11	23	57
Clear cell carcinoma	4	13	17	34	1	1	18	48	0	5	11	52
Papillary renal cell carcinoma	3	4	2	1	1	5	2	2	0	3	5	2
chromophobe cell carcinoma	5	5	3	0	2	7	3	1	1	3	7	2
Nephroblastic	0	0	1	0	0	0	0	1	0	0	0	1
mucinous carcinoma	1	0	0	0	1	0	0	0	1	0	0	0

CDFI was significantly different from SMI vascular architecture, 2 to 3 was significantly lower than SMI (*x*^*2*^ = 5.557, *P* = 0.018). Using the blood flow grading shown by MFI images in the clearance period as a reference, there was a statistically significant difference between CDFI and MFI in evaluating the blood flow grading of renal masses (*x*^*2*^ = 10.165, *P* = 0.001). There was no statistical significance in blood flow grading between SMI and MFI (*x*^*2*^ = 2.372, *P* = 0.499).

### Vascular architecture analysis of 144 renal lesions based on CDFI, SMI and MFI

**Fig. 3:**
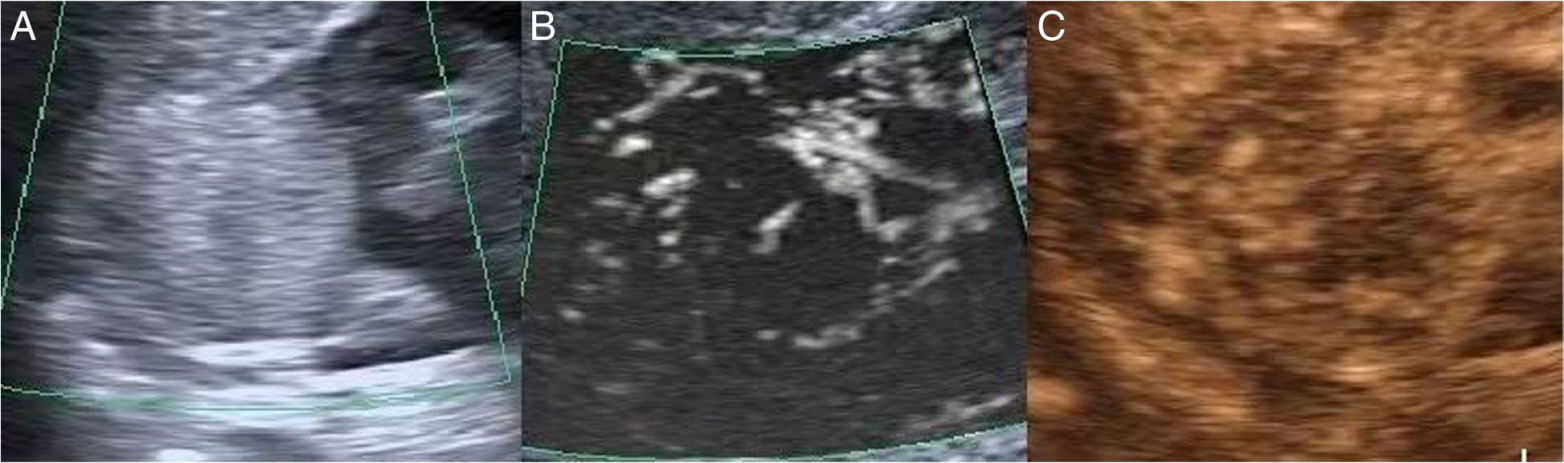
A solid tumor of the right kidney **A** ultrasound imaging, hyperechoic mass with clear boundary, regular shape, and inhomogeneous internal echo **B** SMI show a small blood vessels can be seen in the mass, the blood vessel architecture for type II **C** MFI showed peripheral and internal contorts the blood vessels, blood vessel vascular architecture for type III, pathological results of hamartoma

**Fig. 4:**
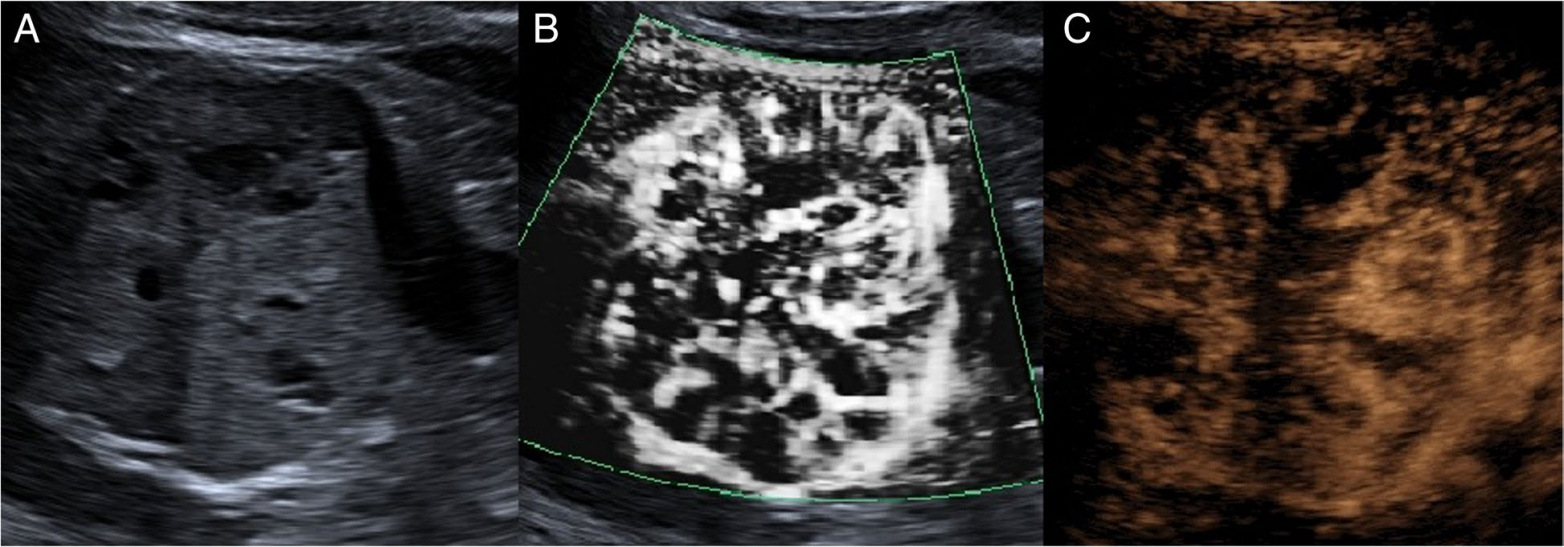
A solid tumor of the right kidney **A** ultrasound imaging, isoechoic mass with unclear boundary, regular shape, and inhomogeneous internal echo, with hypoecho and echoless area and small fluid area **B** SMI the growth from the periphery to the interior was tortuous and disorderly, with multiple branching vessels, and the vascular architecture was type V **C** MFI images showed the tortuous and disorderly growth of multi-branched vessels from the periphery to the interior. The vascular architecture was type V. Pathological results showed clear cell renal cell carcinoma

In Table 3, CDFI, SMI and MFI were used to examine renal masses, vascular architecture of type I, II and III accounted for 75% (108/144), 50.7% (73/144) and 43.1% (62/144), respectively. Type IV and V vascular architecture accounted for 25% (36/144), 49.3% (71/144) and 56.9% (82/144). SMI could display low-speed blood flow signals, thus showed more detailed and accurate delineation of vascular architecture (Figs. 3 and 4). Using the vascular architecture shown in MFI images during clearance period as a reference, the coincidence rates of CDFI and SMI vascular architecture classification were 56.25% (81/144) and 75.69% (109/144), respectively.

**Table 3:** Vascular architecture of 144 renal lesions based on CDFI, SMI and MFI

	CDFI					SMI					MFI				
	I	II	III	IV	V	I	II	III	IV	V	I	II	III	IV	V
Benign	18	17	13	3	0	11	12	23	3	2	7	14	24	3	3
angiomyolipomas	17	16	11	2	0	10	12	22	2	0	6	14	23	2	1
eosinophilic adenomas	0	1	2	1	0	0	0	1	1	2	0	0	1	1	2
inflammatory pseudotumor	1	0	0	0	0	1	0	0	0	0	1	0	0	0	0
Malignance	13	17	30	22	11	5	9	13	26	40	2	4	11	29	47
Clear cell carcinoma	5	11	24	17	11	1	2	8	18	39	0	0	7	19	42
Papillary renal cell carcinoma	3	2	4	1	0	1	3	3	5	1	0	2	2	5	4
chromophobe cell carcinoma	4	4	2	3	0	2	4	2	2	0	1	2	2	5	0
Nephroblastic	0	0	0	1	0	0	0	0	1	0	0	0	0	0	1
mucinous carcinoma	1	0	0	0	0	1	0	0	0	0	1	0	0	0	0

CDFI was significantly different from SMI vascular architecture, IV and V was significantly lower than SMI(*x*^*2*^ = 18.217, *P* < 0.001). Using the vascular architecture of MFI images in the clearance period as a reference, there was a statistically significant difference between CDFI and MFI in the evaluation of renal tumor vascular architecture (*x*^*2*^ = 29.518, *P* < 0.001). There was no statistically significant difference between SMI and MFI in the evaluation of renal tumor vascular architecture (*x*^*2*^ = 3.048, *P* = 0.550).

### The diagnostic performance of CDFI, SMI and MFI

**Fig. 5:**
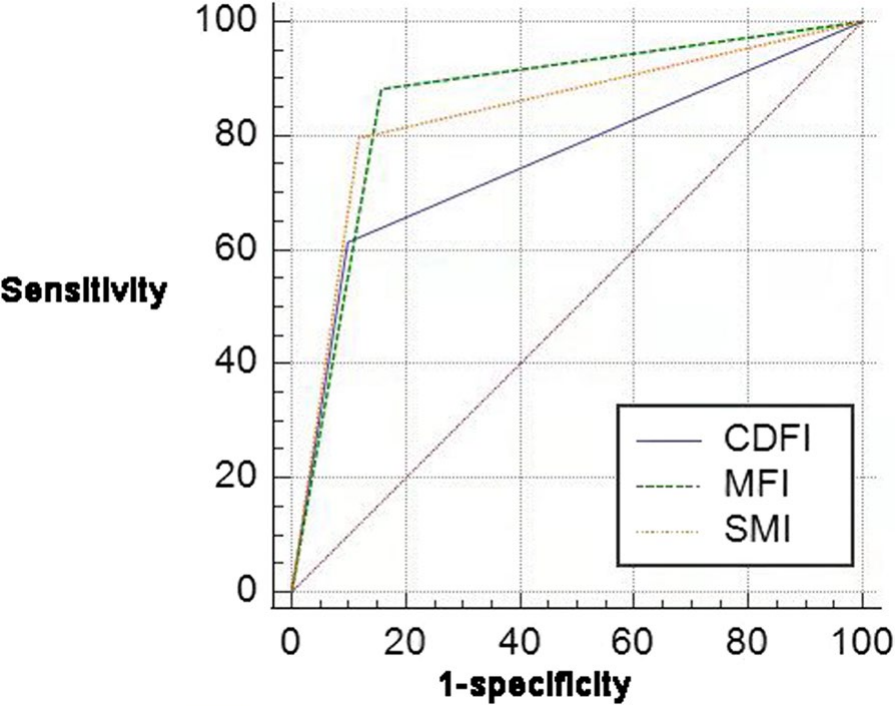
The ROC curves of identify benign and malignant renal lesions in CDFI. SMI and MFI

The diagnosis of benign lesions was based on blood flow grade 0, grade 1, and vascular architecture pattern type I, II and III. The diagnosis of malignant lesions was based on blood flow grade 2, 3 and vascular architecture type IV, V. The diagnostic results of CDFI, SMI and MFI were shown in Table 4. The sensitivity, specificity, 95% confidence interval and area under the ROC curve of SMI and MFI for the diagnosis of benign and malignant renal lesions were calculated respectively, as shown in Table 5. The areas under the ROC curves(AUC) of the three groups were 0.757, 0.839, and 0.862, respectively. Z test results showed that there was a statistically significant difference in the diagnostic efficiency between CDFI and MFI in benign and malignant diagnosis of renal masses (*Z* = 3.687, *P* = 0.0002). The diagnostic efficacy of SMI and MFI in the diagnosis of benign and malignant kidney was not statistically significant (*Z* = 1.167, *P* = 0.2431), Fig. 5.

**Table 4:** CDFI, SMI and MFI in the diagnosis result of benign and malignant renal tumors

Pathological type	CDFI		SMI		MFI	
	malignant	benign	malignant	benign	malignant	benign
malignant	57	36	74	19	82	11
benign	5	46	6	45	8	43

**Table 5:** The diagnostic performance of CDFI, SMI, MFI

	Sensitivity	Specificity	95% CI ^b^	Area under ROC Curve
CDFI	61.29	90.20	0.679–0.825	0.757
SMI	79.57	88.24	0.769–0.895	0.839
MFI	88.17	84.31	0.795–0.914	0.862

## Discussion

Contrast-enhanced ultrasound micro-flow imaging (MFI) is a new contrast-enhanced ultrasound imaging technology. This technology uses ultrasound with high output power to crush the contrast agent microbubbles, tracks and traces the reperfusion movement trajectory of the contrast agent under low mechanical index ultrasound, and superimposes the images at different time points, so as to clearly display the micro-vascular network [5].

SMI technology has developed rapidly in recent years, it can distinguish between blood flow and the noise of tissues motion, using the calculation method of the adaptive show true blood flow information, reduce motion artifacts, clear display of tiny, low speed of blood flow signals, can improve the diagnostic sensitivity and specificity, and do not need to inject contrast agents, belongs to the noninvasive examination. At present, it has been applied in the study of breast, thyroid and liver diseases [5–9].

Neovascularization is very important for tumor growth and proliferation [10]. Renal carcinoma can secrete a variety of vasoactive substances, induce the formation of a large number of new blood vessels and form a rich microvascular network. Therefore, the number, structure and distribution of new blood vessels in benign and malignant renal masses are obviously different. Angiography showed that there were new blood vessels in more than 90% of renal cancer lesions, which were mostly densely clustered [11]. The microvascular structure of different parts of renal carcinoma was observed by transmission electron microscopy. It was found that the density of vessels was the largest in the lateral part near the tumor tissue, and a large number of vessels were connected with each other through anastomosis. In the central area, hemoperfusion is relatively insufficient, blood vessels are disorderly and irregularly distributed, and there are necrotic foci of different shapes and sizes in this area [12]. In this study, through SMI and MFI observation, it was found that the renal malignant tumor had rich blood flow signals, and there were thick blood vessels around the tumor, and the blood vessels gradually extended inward from the periphery, accompanied by multiple branches. The blood vessels were tortuous and disorderly, irregular expansion and interruption could be seen, which was dendritic, and the continuation of normal renal blood flow was lost. The blood flow grade was mainly III, and the vascular architecture was mainly IV (global flow type) and V (multi-flow type). There was no statistical significance between SMI and MFI in evaluating the vascular architecture and blood flow grade of renal benign and malignant tumors, indicating that the two methods had similar ability to display blood flow information of renal tumors. In the malignant tumor group, SMI examination of 3 tumors showed no obvious blood flow signal, and MFI showed fine vessels extending from the periphery to the center. The maximum diameter of two tumors was less than 2 cm, and the location of one tumor was too deep, which contributed to being incapable of SMI in showing the blood flow. Therefore, MFI was better than that of SMI in the examination of the largest diameter less than 2 cm, deep location or dorsal renal masses. In the malignant tumor group, there were 79 tumors with IV and V vascular architectures detected by SMI, and 85 tumors were detected by MFI, indicating that the display ability of SMI was slightly inferior to that of MFI for small branch vessels at the end of new vessels.

Renal benign neoplasm blood signal is relatively less prominent as compared to malignant neoplasm, blood flow grade 0, 1, blood vessel architecture of type I (no flow), type II (dot) and III (less blood flow type). Dot, rod-shaped and evenly distributed gentle blood vessels with equal thickness [13]. In the benign tumor group, SMI determined 5 tumors with IV-V vascular architecture, and 8 tumors with blood flow grade above grade 2. MFI determined the vascular architecture of 6 masses as IV-V, and the blood flow grade of 10 masses was above grade 2. The combined analysis of vascular architecture and blood flow grade showed that a total of 5 masses showed large peripheral and internal blood flow and abundant blood flow, including 4 cases of hamartoma and 1 case of eosinophilic adenoma. Hamartoma is composed of blood vessels, smooth muscle and fat. When the blood vessels lack elastic layer and are closed and incomplete, the distorted and deformed vascular network can be seen in the tumor, and the blood sinusoid is easily formed in the tumor. Among the 3 cases of hamartomas, the largest diameter of 1 case was 13.3 cm, and the formation of blood sinuses could be seen in the gross specimen mass. However, eosinophilic adenoma is a very rare benign tumor, which is mostly characterized by rich blood supply. The enhanced CT examination in the cortical phase shows uneven enhancement, and the typical case shows spoke-wheel enhancement [14].

CDFI, SMI and MFI can all display the blood flow information in the renal mass. Different observers have good consistency in the evaluation of the vascular architecture and blood flow classification of the three examination methods. CDFI is inferior to SMI and MFI in the evaluation of blood flow classification and vascular architecture of renal masses.

SMI is a non-invasive, safe and relatively economical examination, which can realize real-time observation of multiple sections of lesions. MFI requires injection of contrast agent and can only be observed on a single section at a time. In terms of blood flow signal detection, SMI can clearly display the shape of blood vessels in the lesion and directly observe the vascular diameter, while MFI can detect more blood flow signals by reflecting the microbubble trajectory. The blood flow display ability of SMI was still inferior to that of MFI for deep lesions.

## Conclusion

SMI can display low-speed blood flow, clearly display blood flow information within the lesion, and improve the detection of blood flow information in renal masses, which has a good clinical application value.

## Data Availability

The datasets used during the current study are available from the corresponding author on reasonable request.

## Abbreviations

CDFI: Color Doppler Flow Imaging
SMI: Superb Microvascular Imaging
MFI: Contrast-enhanced Ultrasound Microflow Imaging
SF6: Sulfur hexafluoride
